# Age Friendly Health System 4M Competency-Based Curriculum for Internal Medicine Residents

**DOI:** 10.1093/geroni/igaf122.3151

**Published:** 2025-12-31

**Authors:** Tiffany Hu, Cecily McIntyre, Gregory Ouellet, Marcia Mecca, Jennifer Ouellet

**Affiliations:** Yale School of Medicine, New Haven, Connecticut, United States; Yale School of Medicine, New Haven, Connecticut, United States; Yale School of Medicine, New Haven, Connecticut, United States; Yale School of Medicine, New Haven, Connecticut, United States; Yale School of Medicine, New Haven, Connecticut, United States

## Abstract

The Age Friendly Health System 4Ms is an evidence-based framework to provide high value care to older adults, with which educational content in Geriatrics is increasingly aligned. Focusing on Internal Medicine residents, who provide care to older adults in a variety of settings, we aimed to assess perceived proficiency in core competencies, as identified by AGS/ADGAP and organized using the 4Ms framework. We surveyed Internal Medicine residents in three programs at one academic medical center. The survey asked residents about their self-reported proficiency in AGS/ADGAP competencies on a scale of 1-5 (1 “completely unable to perform this skill”, 5 “I am expert and can teach this skill to others”). A total of 35 residents responded to the pre-curriculum survey, including 13 PGY1, 17 PGY2 and 5 PGY3 residents. Most (71%) had no Geriatrics-focused experience in medical school, yet 27 individuals (77%) had >1 Geriatrics rotation during residency. Residents self-reported lower proficiency scores in competencies related to mobility (3.21) and multicomplexity (3.39). Of the competencies, residents scored lowest in their ability to screen for pressure injuries (2.95) and develop a multifaceted plan for fall prevention (3.00). Our findings highlight gaps in Internal Medicine residents’ self-reported proficiency in key Geriatrics competencies. These results will inform our development of a QR-generated skills tracker for residents to have skills in Geriatrics evaluated in real time by trained faculty. This will also help align curricular content in didactics and rotations to target highest priority learning needs.

